# Cyclin D1 and Ki-67 expression correlates to tumor staging in tongue squamous cell carcinoma

**DOI:** 10.4317/medoral.20601

**Published:** 2015-10-09

**Authors:** Eduardo-Pereira Guimarães, Marina-Lara de Carli, Felipe-Fornias Sperandio, João-Adolfo-Costa Hanemann, Alessandro-Antônio-Costa Pereira

**Affiliations:** 1 DDS, MSc. DDS, PhD. Department of Clinic and Surgery, School of Dentistry, Federal University of Alfenas, Alfenas, Minas Gerais, Brazil; 2 DDS, PhD. DDS, PhD. Department of Pathology and Parasitology, Institute of Biomedical Sciences, Federal University of Alfenas, Alfenas, Minas Gerais, Brazil

## Abstract

**Background:**

The immunohistochemical expression of Cyclin D1 and Ki-67 were analyzed in tongue squamous cell carcinomas (SCC), relating them to the clinical and morphological exhibition of these tumors.

**Material and Methods:**

Twenty-nine patients fulfilled the inclusion criteria; clinical data included gender, age, ethnicity and use of licit drugs such as alcohol and tobacco. The TNM staging and histopathological differentiation grading was assessed for each case. In addition, T1 patients were gathered with T2 patients; and T3 patients were gathered with T4 patients to assemble two distinct groups: (T1/T2) and (T3/T4).

**Results:**

The mean follow-up time was 24 months and 30% of the patients died as a consequence of the disease, while 23.3% lived with the disease and 46.7% lived lesion-free. T1 and T2 tumors showed statistically lesser Ki-67 and Cyclin D1 staining when compared to T3 and T4 tumors.

**Conclusions:**

Ki-67 and Cyclin D1 pose as auxiliary tools when determining the progression of tongue SCC at the time of diagnosis.

**Key words:**Carcinoma, squamous cell, cyclin D, immunohistochemistry, Ki-67 antigen, prognosis.

## Introduction

Oral squamous cell carcinoma (SCC) is responsible for a high mortality rate of 3 to 10% worldwide (1). The great majority of oral SCC is diagnosed late, what poses as one of the main factors that compromise patients` survival rates, though the early detection of this lesion is in fact increasing (2). In that way, almost 50% of oral SCC cases diagnosed between 2002 and 2008 in the United States presented regional consequences instead of only local commitment (1).

The great majority of oral malignant tumors consist on tongue SCCs, which mainly affect middle age men (1); in fact, patients older than 65 can correlate to worse prognosis of T1 and T2 (tumor size - TNM classification) tongue SCCs (3). The TNM staging is widely used to predict the clinical behavior of tumors (4). However, T staging alone does not envisage the local recurrence or distant spreading of the tumor (5); accordingly, several demographic and histopathological features have been closely related to the TNM system (3).

Higher T stages along with Ki-67 over expression can in fact be linked to loco-regional recurrence of oral and oropharynx SCCs (6); and among the genetic aberrations frequently seen in tongue SCC, Cyclin D1 super expression can surely be mentioned besides Ki-67 (7,8), though the exact interference of these genetic mutations in the transition between a normal to a neoplastic epithelium is still yet to be established (9). A single histopathological specimen may hold precious information about the comportment of the lesion from which this specimen originated.

Although the early detection of oral SCC is eventually turning into reality (2) along with advances in cancer prevention or treatment, the five-year survival rate of a patient diagnosed with head and neck SCC remains poorer than those of other cancers, such as cervix, breast and colorectal (10). Actually, the survival rates of tongue SCC have not been improved over the years (11), and oral cancer rates have become higher in some countries (12); all of these reflect the need for studies to check the current incidence and clinical behavior of these lesions. Moreover, there is actually no established marker to routinely predict the prognosis of head and neck SCC (13); besides, the mechanisms underlying the malignant progression of head and neck SCCs remains unclear (14). Thus, this study aimed to evaluate the expression of Cyclin D1 and Ki-67 within tongue SCCs to correlate this expression to the clinical appearance and behavior of the tumors.

## Material and Methods

The authors wish to declare that all experiments on human subjects were conducted in accordance with the Declaration of Helsinki; all procedures were carried out after the adequate understanding and written consent of the subjects. The study was only carried out after obtaining approval from the Human Subjects Ethics Committee of our institution; protocol number 358.828.

The medical records of 30 patients diagnosed with oral SCC located exclusively in the tongue were randomly collected from a regional Oncology Center between 2006 and 2013. The clinical data included gender, age, ethnicity and use of licit drugs such as alcohol and tobacco. The TNM staging, as well as lesion type (Exophytic or infiltrative growth or not described), treatment realized (surgery, radiotherapy, chemotherapy), compromised lymph nodes, time to recurrence (if any) and current situation of the patient were also included in the evaluation of the files. In addition, T1 patients were gathered with T2 patients and T3 patients were gathered with T4 patients to assemble two distinct groups: (T1/T2) and (T3/T4).

The patients were followed from the date they received treatment until the patient`s death or through an average of 24.14 months. Clinical specimens of the tumors were collected from all the patients, fixed in formaldehyde, and routinely processed until paraphin inclusion for morphological and immunohistochemical analyses; 3µm-thick slides were stained with hematoxylin and eosin (HE) and proceded to morphological evaluation. Complete specimens for all the cases who underwent resection were obtained. Regarding the non-resectable cases, biopsies were obtained for the immunohistochemical procedures.

Oral SCC diagnoses were revised by two expert pathologists and were classified into two groups according to Bryne *et al*. (15): WD = well diferentiated SCC; MD = moderately diferentiated SCC; and PD = poorly differentiated SCC. After diagnostic confirmation the cases followed immunohistochemical reactions with anti-Cyclin D1 (SP4 clone Rabbit Monoclonal, ref 100419, Spring, Pleasanton, CA, USA) and anti-Ki-67 (MIB-1 clone Rabbit Monoclonal, ref 56534, Dako, Glostrup, Denmark).

The immunohistochemical staining was performed through the standard streptavidin-biotin-peroxidase complex (StreptABComplex/HRP, Duet Mouse/Rabbit, ref K0492, Dako, Glostrup, Denmark). After antigen retrieval assured by specimen incubation with 10mM of Citric Acid buffered solution), pH 6.0, the endogenous peroxidase was quenched by incubation in 3% hydrogen peroxide in methanol (1:1) for 15 minutes at room temperature. Slides were then incubated overnight at 4oC with the previously mentioned antibodies and at the following dilutions: 1:400 and 1:300 for Cyclin D1 and Ki-67, respectively.

The slides were counter-stained with Harris hematoxylin before serial hydration. For both biomarkers a positive control was represented by a tonsil fragment, and as negative control, a representative slide of the same tissue, however lacking the primary antibody was used.

To quantify all samples through immunohistochemical staining 10 fields were analyzed (107.040.116 μm2) for each biomarker (16) in a 400 magnification utilizing an optic microscope (AxioLab Al Plus, Carl Zeiss, Jena, Germany). Images were then captured with the help of an appropriate camera (AxioCam MRc, Carl Zeiss, Jena, Germany) and examined with special software (AxioVision Rel 4.8.2, Carl Zeiss, Jena, Germany). The analysis was performed in 10 fields of the invasive front for all cases. All cells that exhibited brownish stained nuclei were considered positive, independently of the color intensity; all positive cells were counted in each field and the mean of all 10 fields was obtained for each field (6). One case was excluded from the immunohistochemical analysis due to insufficient material.

First, to verify the differences between the patients` mean ages the Student’s t-test was used and, subsequently, the age variable was categorized. The Fisher exact test or chi-square test was used to assess the association among demographic, clinical and microscopic variables, with a significance level of 5%. Only patients who underwent resection were included to verify association between recurrence and the expression of the biomarkers.

According to Wangsa *et al*. (6), to verify the associations between immunostaining and clinicopathological characteristics a cut-off point was statistically decided for each immunohistochemical biomarker staining considering the median: 19 for Cyclin D1 and 28 for Ki-67. The software utilized to perform all the statistical analyses was STATA 7.0 (StataCorp LP, College Station, TX, USA).

## Results

White men prevailed among the studied subjects with a mean age of 54.6 years. Among the lesion types we found 23 with an infiltrative growth (76.6%); 5 with exophytic growth (16.7%) and 2 not described in the respective medical files (6.7%). Smokers and alcoholics represented 70% of all collected data. Regarding TNM staging, 08 (26.6%) patients were classified as T1, 11 (36.6%) as T2, 04 (13.3%) as T3 and 07 (23.3%) patients were classified as T4.

Treatment modalities included surgery alone for 3 (10%) patients; surgery with neck dissection followed by radiotherapy for 9 (30%) patients; surgery without neck dissection followed by radiotherapy for 3 (10%) patients; surgery with and without neck dissection followed by adjuvant radio chemotherapy for 7 (23.3%) and 1 (3.3%) patient, respectively. All 7 patients (23.3%) who did not undergo surgical excision received concurrent radio chemotherapy.

The mean follow-up time was 24.14 months, varying between 5 to 81 months. During follow-up 12 subjects (40%) presented lesion recurrence, being 4 and 8 patients from (T1/T2) and (T3/T4) groups, respectively. Interestingly, (T3/T4) group presented significantly more lesion re-incidence (*p*= 0.009) than the (T1/T2). The local recurrence was the most frequent (41.6%). Recurrence mean time was 11.8 months, varying between 1.2 to 21.7 months after the initial diagnosis. At the end of the follow up period 18 patients (60%) were alive without any recurrence, and among the 6 non-resectable patients 5 (83.3%) presented an active illness. Moreover, 6 (54.5%) patients from the (T3/T4) group died, whilst only 3 (15.8%) patients from (T1/T2) group deceased.

**Figure 1:**
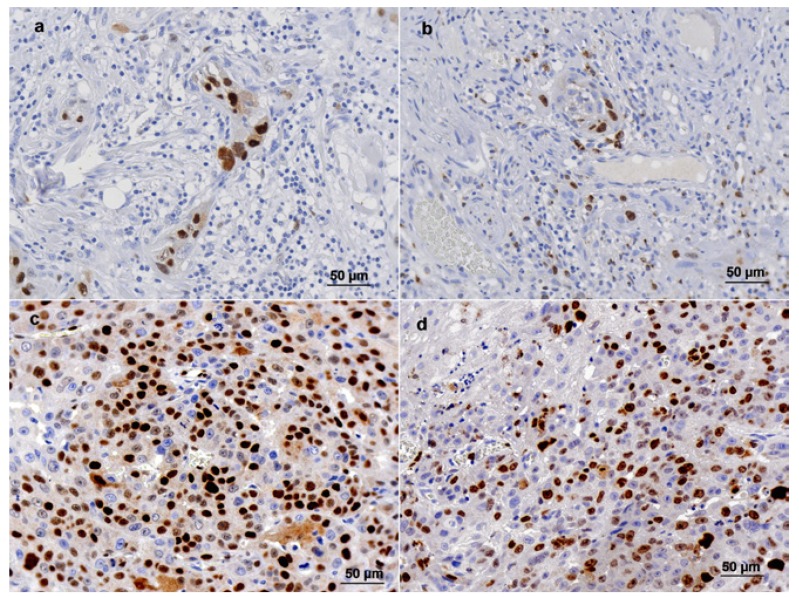
Cyclin D1 and Ki-67 immunohistochemical staining in tongue squamous cell carcinoma. a and b - Cyclin D1 staining (≤ 19 stained cells per field) and Ki-67 staining (≤ 28 stained cells per field) in the same tumor, respectively; c and d - Cyclin D1 staining (> 19 stained cells per field) and Ki-67 staining (> 28 stained cells per field) in the same tumor respectively. 400X magnification.

Cyclin D1 positive cells varied from 0 to 136 in each counted field (mean of 31.7 and median of 19.5) (Fig. 1a, c). Cyclin D1 immunostaining was less than 39.5 cells per field in 21 cases (72.4%); its expression showed a statistically significant relation with T staging (*p*= 0.02), but there was not a statistically significant association with gender, age, ethnicity, type of lesion, N staging, histopathological differentiation, involved lymph nodes or recurrence ( Table 1).

**Table 1:**
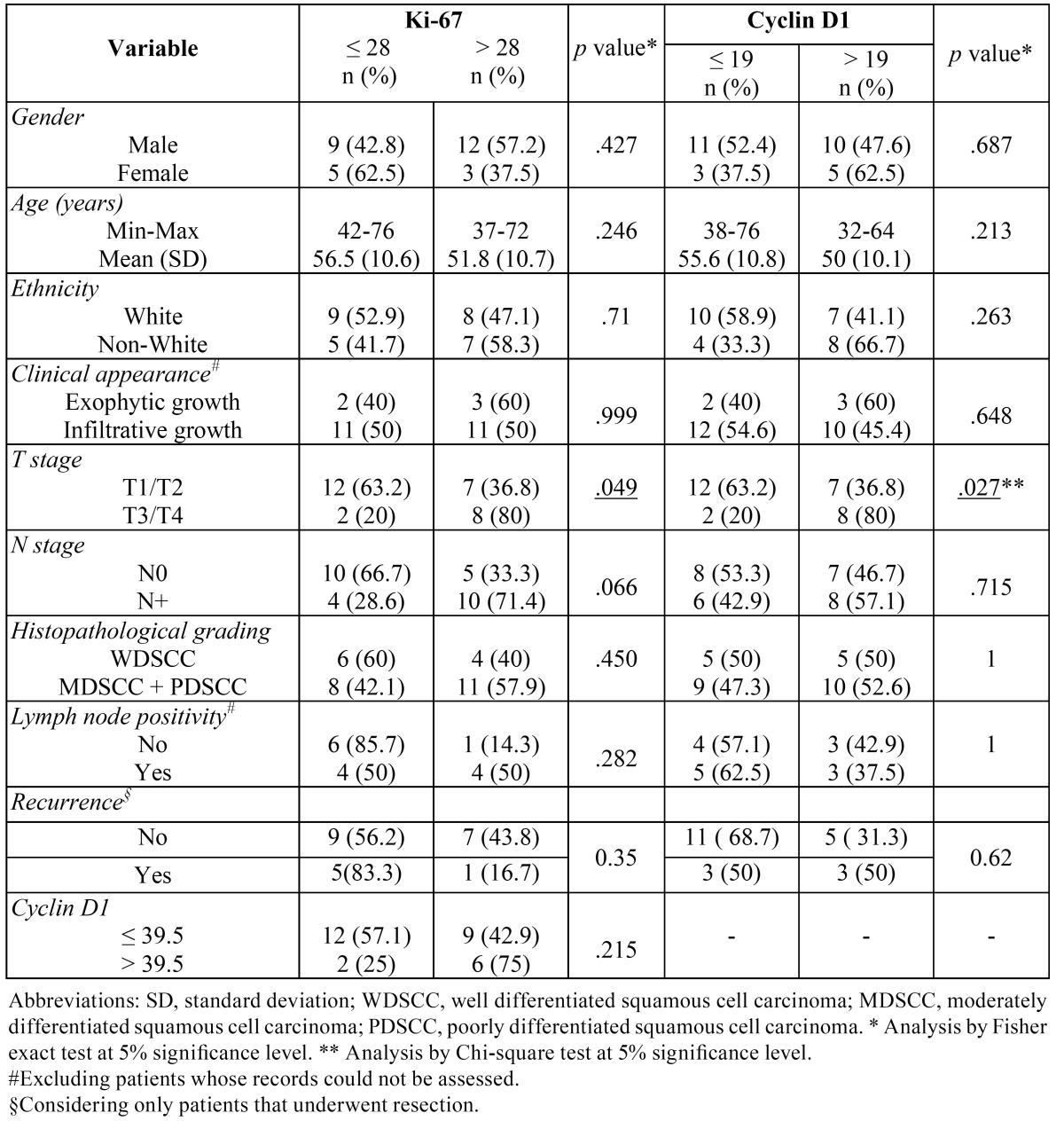
Clinical and histopathological features of tongue squamous cell carcinomas relating to Ki-67 and cyclin D1 immunostaining.

Regarding Ki-67, stained cell counting varied from 0 to 214.4 (mean of 44.4 and median of 28.4) (Fig. 1 b, d). Its expression showed the same prevalence (50%) of patients for both higher than and lesser than the cutoff point {28}. Ki-67 staining was also statistically associated to the T staging (0.049); besides,  Table 1 shows a possible trend of statistical connection between Ki-67 positivity and the N staging of tumors (*p*= 0.06). Nevertheless, there was not a statistically significant association with gender, age, ethnicity, type of lesion, histopathological differentiation, involved lymph nodes or recurrence ( Table 1).

## Discussion

Most oral SCC arise from tongue in men older than 50 years of age (1,17), and a significant increase of this lesion in patients over 80 years-old was observed lately, probably due to a higher life expectancy (2). There is also a classic ratio between men and women of 3.5:1 or 3:1 to present this cancer, while novel reports reveal a more equal leveling between the genders (1,17); however, our study is in accordance with the majority of published literature, disclosing 70% of male patients and a gender proportion of 2.33 men to each woman (mean age of 54.63 years).

Nonetheless, the raise of tongue SCC in women can be explained by behavioral changes along the past years; accordingly, tabagism and etilism were encountered as routines for the majority of men and women (76.7 and 70%, respectively), what surely reassures these habits as strong risk factors to promote oral SCC (12,18,19).

With respect to TNM classification Group T3/T4 was statistically connected to lesion recurrence, what poses as an indeed interesting factor to guide clinical conduct. Consonantly to our study, previous reports point the advanced T4 staging and committed lymph nodes as predictors of a less favorable prognosis (1,17,20). Nevertheless, as already mentioned T staging solely does not envisage the local recurrence or distant spreading of the tumor (5) and that explains the urge to correlate demographic and histopathological features to the TNM system (3).

In fact, biomarkers have been widely utilized to elucidate the clinical behavior of oral SCCs (21,22), mainly Cyclin D and Ki-67 proteins (6,17,23). Cyclin D is a cell cycle regulator that binds to cyclin-dependent kinase (CDK) 4 and CDK6. This cyclin-CDK complex plays a key role on the induction of DNA synthesis and transition of cells between G1/S phases (24), making it a true target during carcinogenesis (25). Accordingly, cyclin D1 amplification has been recognized in 30% of head and neck SCC (26). Consonantly, NF-κB signaling pathway may play an important role at the early stages of carcinogenesis; its levels increase gradually from pre-malignant lesions to invasive head and neck SCC (27,28); in accordance, the over expression of NF-κB leads to the activation of growth promoting genes such as c-myc and Cyclin D1 (29,30).

Moreover, an elevated Cyclin D1 expression has been associated with higher tumor size, lymph node commitment and poor prognosis (17,31-33); interestingly, we have seen that invasive clones of a tongue SCC cell line presented increased levels of cyclin D1 when compared to its noninvasive counterpart (7). Accordingly, cyclin D1 is often a target when treating oral cancer (34). A higher level of dysplasia in oral SCCs and pre-malignant lesions was also previously related to Cyclin D1 over expression (31,35,36). Suitably, the present study showed that 80% of T3/T4 cases had significantly higher Cyclin D1 and Ki-67 expression, what serve as a useful link between the clinical and histopathological appearance of tongue SCCs.

Regarding Ki-67, it is a current nuclear antigen in S, G2, and M phase, i.e., it presents throughout the cell cycle, which offers a reliable outcome of the growth rate of neoplastic human cells (37). In oral normal epithelium, low levels of Ki-67 expression have been found while normal appearing mucosal epithelium bordering the cancerous lesions has demonstrated low to moderate levels of this marker (38). Several studies verified the possible association between highly expressed Ki-67 and dysplasia degrees, for instance (39,40); and a recent study demonstrated that CD147 and Ki-67 overexpression is linked to a worse prognosis of tongue SCC (41). Remarkably, Sittel *et al*. (42) mentioned that tumors widely stained with Ki-67 respond better to radiation therapy, what is understandable since highly proliferative cells are more sensible to radiation (43).

Although Martinez *et al*. (44) failed to demonstrate a statistical difference of Ki-67 expression between normal and dysplastic mucosa of the lips, Wangsa *et al*. (6) stated that a high-proliferative activity encountered with Ki-67 staining relates to an elevated risk of recurrence in tongue SCCs. An interesting recent study also showed that Ki-67 expression in mucosal areas distant from oral SCCs serves as a prognostic factor for these lesions (45).

The fact that we had no data on the clinical appearance of the lesions for two patients poses as a limitation to the current study. Actually, retrospective studies often present incomplete clinical data. Moreover, the reason why some patients, but not others, underwent neck dissection remains unclear (46), once lymph node positivity at diagnosis was not described for fourteen patients. Secondly, immunohistochemical staining is widely used in OSCC (6,17,47,48); however, this method allows for the evaluation of individual molecules, while cancer biology involves the interaction of some distinct molecules and pathways (49). Moreover, this technique depends on sufficient paraffin specimens to be performed.

In conclusion, tumors presenting higher T stages and poor prognosis also presented higher Cyclin D1 and Ki-67 staining, what enforces these biomarkers as an auxiliary tool to predict the progression of tongue SCC at the time of diagnosis.

## References

[1] Siegel R, Naishadham D, Jemal A (2013). Cancer statistics, 2013. CA Cancer J Clin.

[2] Marocchio LS, Lima J, Sperandio FF, Correa L, de Sousa SO (2010). Oral squamous cell carcinoma: an analysis of 1,564 cases showing advances in early detection. Journal of oral science.

[3] Kantola S, Parikka M, Jokinen K, Hyrynkangs K, Soini Y, Alho OP (2000). Prognostic factors in tongue cancer - relative importance of demographic, clinical and histopathological factors. British journal of cancer.

[4] van der Schroeff MP, Baatenburg de Jong RJ (2009). Staging and prognosis in head and neck cancer. Oral oncology.

[5] Nixon IJ, Palmer FL, Lakin P, Kattan MM, Lee NY, Ganly I (2013). Pathologically determined tumor volume vs pathologic T stage in the prediction of outcome after surgical treatment of oropharyngeal squamous cell carcinoma. JAMA otolaryngology-- head & neck surgery.

[6] Wangsa D, Ryott M, Avall-Lundqvist E, Petersson F, Elmberger G, Luo J (2008). Ki-67 expression predicts locoregional recurrence in stage I oral tongue carcinoma. British journal of cancer.

[7] Giudice FS, Dal Vechio AM, Abrahao AC, Sperandio FF, Pinto-Junior Ddos S (2011). Different expression patterns of pAkt, NF-kappaB and cyclin D1 proteins during the invasion process of head and neck squamous cell carcinoma: an in vitro approach. Journal of oral pathology & medicine : official publication of the International Association of Oral Pathologists and the American Academy of Oral Pathology.

[8] Sperandio FF, Giudice FS, Corrêa L, Pinto DS, Hamblin MR, de Sousa SC (2013). Low-level laser therapy can produce increased aggressiveness of dysplastic and oral cancer cell lines by modulation of Akt/mTOR signaling pathway. J Biophotonics.

[9] Goessel G, Quante M, Hahn WC, Harada H, Heeg S, Suliman Y (2005). Creating oral squamous cancer cells: a cellular model of oral-esophageal carcinogenesis. Proceedings of the National Academy of Sciences of the United States of America.

[10] Jemal A, Siegel R, Ward E, Hao Y, Xu J, Murray T (2008). Cancer statistics, 2008. CA Cancer J Clin.

[11] Lam L, Logan RM, Luke C (2006). Epidemiological analysis of tongue cancer in South Australia for the 24-year period, 1977-2001. Australian dental journal.

[12] Warnakulasuriya S (2009). Global epidemiology of oral and oropharyngeal cancer. Oral oncology.

[13] Bello IO, Soini Y, Salo T (2010). Prognostic evaluation of oral tongue cancer: means, markers and perspectives (I). Oral oncology.

[14] Bagan JV, Scully C (2008). Recent advances in Oral Oncology 2007: epidemiology, aetiopathogenesis, diagnosis and prognostication. Oral oncology.

[15] Bryne M, Koppang HS, Lilleng R, Stene T, Bang G, Dabelsteen E (1989). New malignancy grading is a better prognostic indicator than Broders' grading in oral squamous cell carcinomas. J Oral Pathol Med.

[16] Odajima T, Sasaki Y, Tanaka N, Kato-Mori Y, Asanuma H, Ikeda T (2005). Abnormal beta-catenin expression in oral cancer with no gene mutation: correlation with expression of cyclin D1 and epidermal growth factor receptor, Ki-67 labeling index, and clinicopathological features. Human pathology.

[17] Carlos de Vicente J, Herrero-Zapatero A, Fresno MF, López-Arranz JS (2002). Expression of cyclin D1 and Ki-67 in squamous cell carcinoma of the oral cavity: clinicopathological and prognostic significance. Oral Oncol.

[18] Petersen PE (2009). Oral cancer prevention and control--the approach of the World Health Organization. Oral oncology.

[19] Petti S (2009). Lifestyle risk factors for oral cancer. Oral oncology.

[20] Massano J, Regateiro FS, Januario G, Ferreira A (2006). Oral squamous cell carcinoma: review of prognostic and predictive factors. Oral surgery, oral medicine, oral pathology, oral radiology, and endodontics.

[21] Henriques AC, de Matos FR, Galvao HC, Freitas Rde A (2012). Immunohistochemical expression of MMP-9 and VEGF in squamous cell carcinoma of the tongue. Journal of oral science.

[22] Mohtasham N, Mahdavi-Shahri N, Salehinejad J, Ejtehadi H, Torabi-Parizi M, Ghazi N (2010). Detection of nucleoproteins in squamous cell carcinoma, and dysplastic and normal mucosa in the oral cavity by methyl green-pyronin staining. Journal of oral science.

[23] Marsit CJ, Black CC, Posner MR, Kelsey KT (2008). A Genotype-Phenotype Examination of Cyclin D1 on Risk and Outcome of Squamous Cell Carcinoma of the Head and Neck. Clin Cancer Res.

[24] Chin D, Boyle GM, Theile DR, Parsons PG, Coman WB (2004). Molecular introduction to head and neck cancer (HNSCC) carcinogenesis. British journal of plastic surgery.

[25] Diehl JA (2002). Cycling to cancer with cyclin D1. Cancer Biol Ther.

[26] Namazie A, Alavi S, Olopade OI, Pauletti G, Aghamohammadi N, Aghamohammadi M (2002). Cyclin D1 amplification and p16(MTS1/CDK4I) deletion correlate with poor prognosis in head and neck tumors. The Laryngoscope.

[27] Mishra A, Bharti AC, Varghese P, Saluja D, Das BC (2006). Differential expression and activation of NF-kappaB family proteins during oral carcinogenesis: Role of high risk human papillomavirus infection. International journal of cancer Journal international du cancer.

[28] Ondrey FG, Dong G, Sunwoo J, Chen Z, Wolf JS, Crowl-Bancroft CV (1999). Constitutive activation of transcription factors NF-(kappa)B, AP-1, and NF-IL6 in human head and neck squamous cell carcinoma cell lines that express pro-inflammatory and pro-angiogenic cytokines. Mol Carcinog.

[29] Reich NC, Liu L (2006). Tracking STAT nuclear traffic. Nature reviews Immunology.

[30] Salgueiredo-Giudice F, Correa-Abrahao A, Fornias-Sperandio F, da-Costa-Dal-Vechio AM, dos-Santos-Pinto-Junior D (2012). An in vitro study showing the three-dimensional microenvironment influence over the behavior of head and neck squamous cell carcinoma. Medicina oral, patologia oral y cirugia bucal.

[31] Vieira RAMAR, Minicucci EM, Marques MEA, Marques SA (2012). Actinic cheilitis and squamous cell carcinoma of the lip: clinical, histopathological and immunogenetic aspects. An Bras Dermatol.

[32] Fujii M, Ishiguro R, Yamashita T, Tashiro M (2001). Cyclin D1 amplification correlates with early recurrence of squamous cell carcinoma of the tongue. Cancer Lett.

[33] Wang L, Liu T, Nishioka M, Aguirre RL, Win SS, Okada N (2006). Activation of ERK1/2 and cyclin D1 expression in oral tongue squamous cell carcinomas: relationship between clinicopathological appearances and cell proliferation. Oral oncology.

[34] Abrahao AC, Giudice FS, Sperandio FF, Pinto Junior Ddos S (2013). Effects of celecoxib treatment over the AKT pathway in head and neck squamous cell carcinoma. Journal of oral pathology & medicine : official publication of the International Association of Oral Pathologists and the American Academy of Oral Pathology.

[35] Kovesi G, Szende B (2006). Prognostic value of cyclin D1, p27, and p63 in oral leukoplakia. Journal of oral pathology & medicine : official publication of the International Association of Oral Pathologists and the American Academy of Oral Pathology.

[36] Turatti E, da Costa Neves A, de Magalhaes MH, de Sousa SO (2005). Assessment of c-Jun, c-Fos and cyclin D1 in premalignant and malignant oral lesions. Journal of oral science.

[37] Gerdes J, Lemke H, Baisch H, Wacker HH, Schwab U, Stein H (1984). Cell cycle analysis of a cell proliferation-associated human nuclear antigen defined by the monoclonal antibody Ki-67. Journal of immunology.

[38] Faratzis G, Tsiambas E, Rapidis AD, Machaira A, Xiromeritis K, Patsouris E (2009). VEGF and ki 67 expression in squamous cell carcinoma of the tongue: An immunohistochemical and computerized image analysis study. Oral oncology.

[39] Thomson PJ, Hamadah O, Goodson ML, Cragg N, Booth C (2008). Predicting recurrence after oral precancer treatment: use of cell cycle analysis. The British journal of oral & maxillofacial surgery.

[40] Kumar P, Kane S, Rathod GP (2012). Coexpression of p53 and Ki 67 and lack of c-erbB2 expression in oral leukoplakias in India. Brazilian oral research.

[41] Yu YH, Morales J, Feng L, Lee JJ, El-Naggar AK, Vigneswaran N (2015). CD147 and Ki-67 overexpression confers poor prognosis in squamous cell carcinoma of oral tongue: a tissue microarray study. Oral Surg Oral Med Oral Pathol Oral Radiol.

[42] Sittel C, Ruiz S, Volling P, Kvasnicka HM, Jungehülsing M, Eckel HE (1999). Prognostic significance of Ki-67 (MIB1), PCNA and p53 in cancer of the oropharynx and oral cavity. Oral Oncol.

[43] Vogin G, Foray N (2013). The law of Bergonie and Tribondeau: a nice formula for a first approximation. International journal of radiation biology.

[44] Martinez A, Brethauer U, Rojas IG, Spencer M, Mucientes F, Borlando J (2005). Expression of apoptotic and cell proliferation regulatory proteins in actinic cheilitis. Journal of oral pathology & medicine : official publication of the International Association of Oral Pathologists and the American Academy of Oral Pathology.

[45] Montebugnoli L, Gissi DB, Badiali G, Marchetti C, Cervellati F, Farnedi A (2011). Ki-67 from clinically and histologically "normal" distant mucosa as prognostic marker in early-stage (T1-T2N0) oral squamous cell carcinoma: a prospective study. Journal of oral and maxillofacial surgery : official journal of the American Association of Oral and Maxillofacial Surgeons.

[46] Nguyen DH, Truong PT (2011). A debate on locoregional treatment of the primary tumor in patients presenting with stage IV breast cancer. Expert review of anticancer therapy.

[47] Hanemann JA, Oliveira DT, Nonogaki S, Nishimoto IN, de Carli ML, Landman G (2014). Expression of E-cadherin and beta-catenin in basaloid and conventional squamous cell carcinoma of the oral cavity: are potential prognostic markers?. BMC cancer.

[48] Kato K, Kawashiri S, Yoshizawa K, Kitahara H, Okamune A, Sugiura S (2011). Expression form of p53 and PCNA at the invasive front in oral squamous cell carcinoma: correlation with clinicopathological features and prognosis. Journal of oral pathology & medicine : official publication of the International Association of Oral Pathologists and the American Academy of Oral Pathology.

[49] Lakhani SR, Ashworth A (2001). Microarray and histopathological analysis of tumours: the future and the past?. Nature reviews Cancer.

